# Temporospatial shifts in the human gut microbiome and metabolome after gastric bypass surgery

**DOI:** 10.1038/s41522-020-0122-5

**Published:** 2020-03-13

**Authors:** Zehra Esra Ilhan, John K. DiBaise, Sydney E. Dautel, Nancy G. Isern, Young-Mo Kim, David W. Hoyt, Athena A. Schepmoes, Heather M. Brewer, Karl K. Weitz, Thomas O. Metz, Michael D. Crowell, Dae-Wook Kang, Bruce E. Rittmann, Rosa Krajmalnik-Brown

**Affiliations:** 1 Biodesign Swette Center for Environmental Biotechnology, Arizona State University, Tempe, AZ USA; 2 Biodesign Center for Fundamental and Applied Microbiomics, Arizona State University, Tempe, AZ USA; 3 Mayo Clinic, Division of Gastroenterology, Scottsdale, AZ USA; 4 Biological Sciences Division, Pacific Northwest National Laboratory, Richland, WA USA; 5 William R. Wiley Environmental Molecular Sciences Laboratory, Pacific Northwest National Laboratory, Richland, WA USA; 6 School of Sustainable Engineering and the Built Environment, Arizona State University, Tempe, AZ USA; 7 Present Address: Department of Civil & Environmental Engineering, The University of Toledo, Toledo, OH USA

**Keywords:** Clinical microbiology, Microbial ecology

## Abstract

Although the etiology of obesity is not well-understood, genetic, environmental, and microbiome elements are recognized as contributors to this rising pandemic. It is well documented that Roux-en-Y gastric bypass (RYGB) surgery drastically alters the fecal microbiome, but data are sparse on temporal and spatial microbiome and metabolome changes, especially in human populations. We characterized the structure and function (through metabolites) of the microbial communities in the gut lumen and structure of microbial communities on mucosal surfaces in nine morbidly obese individuals before, 6 months, and 12 months after RYGB surgery. Moreover, using a comprehensive multi-omic approach, we compared this longitudinal cohort to a previously studied cross-sectional cohort (*n* = 24). In addition to the expected weight reduction and improvement in obesity-related comorbidities after RYGB surgery, we observed that the impact of surgery was much greater on fecal communities in comparison to mucosal ones. The changes in the fecal microbiome were linked to increased concentrations of branched-chain fatty acids and an overall decrease in secondary bile acid concentrations. The microbiome and metabolome data sets for this longitudinal cohort strengthen our understanding of the persistent impact of RYGB on the gut microbiome and its metabolism. Our findings highlight the importance of changes in mucosal and fecal microbiomes after RYGB surgery. The spatial modifications in the microbiome after RYGB surgery corresponded to persistent changes in fecal fermentation and bile acid metabolism, both of which are associated with improved metabolic outcomes.

## Introduction

Roux-en-Y gastric bypass (RYGB) is an effective treatment strategy for morbid obesity and its comorbidities, such as diabetes mellitus^1^. Although the precise mechanisms leading to its success remain unclear, RYGB alters hormonal response^2^, energy metabolism^2^, and bile acid circulation^3^ towards weight loss outcomes. Additionally, an increasing number of studies have shown that RYGB alters gut microbiota in humans^4–11^. The composition of the gut microbiota shifts promptly in humans as soon as 1–3 months after surgery^4,5,10^, and those changes have been reported to persist 12 months post-surgery^5,7,10,12^. Additionally, a number of studies^4,5,7,10,11,13–15^ have evaluated the fecal microbiota after RYGB in longitudinal cohorts.

Due to the invasiveness of mucosal microbiome sampling^16^, studies of the human gut microbiome in obesity and after RYGB have relied on fecal samples^4,5,7,8^, which underrepresent the mucosal communities that actively interact with host immune system and epithelial cells^17^. In healthy humans, composition of mucosal and fecal microbiota varies due to differences in local environments^16,18^. The composition of the mucosal microbiota can drastically change in humans during dysbiosis, such as in ulcerative colitis^19^, colorectal cancer^20^, and diabetes^21^ but, to our knowledge, the mucosal microbiome after RYGB in humans has not been characterized longitudinally.

After RYGB, the metabolic products of the gut microbiota exert beneficial effects on host metabolism^22^. For example, butyrate and propionate, which are known to induce satiety in animals^23^ and humans^24^, were in greater concentrations in post-RYGB patients compared to nonsurgical controls^6^. RYGB surgery also increased bile acid concentrations in plasma^3,8,25^ and this increase has been associated with weight loss in rats following RYGB^26^. An increase in propionate and bile acids after RYGB was associated with an increase in hormone peptide tyrosine tyrosine (PYY) in humans and, hence, resolution of diabetes^27^. Finally, RYGB increased the abundance of amino acid degradation products in feces^6,8^. However, these molecules in connection to microbiome have not been evaluated longitudinally in pre-surgical human populations.

In this study, we characterized the temporal and spatial structures of the microbiome and metabolome in humans before and after RYGB surgery, using 16S rRNA amplicon gene sequencing, gas chromatography-mass spectrometry, liquid chromatography-mass spectrometry, and nuclear magnetic resonance spectroscopy. This longitudinal multi-omic approach revealed differences between mucosal and fecal microbial communities and in fecal metabolites in morbidly obese individuals before and after RYGB surgery. Furthermore, we demonstrated comparable findings from this longitudinal cohort to those of a cross-sectional one.

## Results and discussion

### RYGB surgery induced significant weight loss

**Fig. 1:**
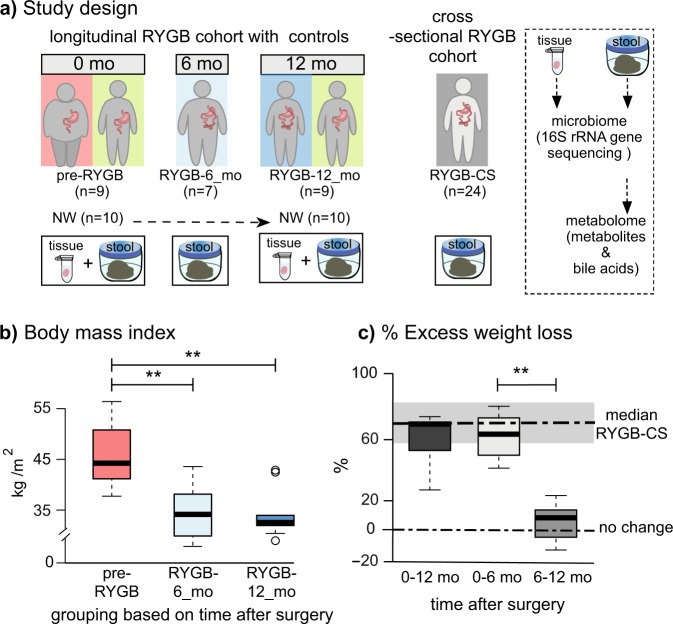
Study design and weight loss after RYGB surgery. **a** Study design including number of participants and sample types collected longitudinally and cross-sectionally. **b** Body mass index (BMI) index of participants before the surgery (pre-RYGB), 6 months (RYGB-6_mo), and 12 months (RYGB-12_mo) after the surgery**. c** % Excess weight loss 12 months after the surgery, 6 months after the surgery, and 6–12 months after the surgery. The box plots represent minimum, maximum, median, first quartile and third quartile values. The gray shaded box around median of RYGB-CS represents median absolute deviation. Statistical significance between the groups was tested with Wilcoxon signed-rank test and *p* values were corrected using the Bonferroni method. **p < 0.01.

We studied the microbiome and metabolome of two cohorts: longitudinal and cross-sectional populations. For the longitudinal arm of the study, we recruited nine morbidly obese pre-RYGB participants (a tenth participant dropped out after baseline measurements) and monitored their weight loss and health outcomes 6 months (RYGB-6_mo) and 12 months (RYGB-12_mo) after RYGB surgery. The study design is presented in Fig. 1a, and participant characteristics are summarized in Table 1. We also compared this longitudinal population to a previously studied cross-sectional RYGB cohort (RYGB-CS) (*n* = 24)^6^.

**Table 1:** Participant characteristics of the longitudinal cohort.

	Pre-RYGB	RYGB-6_mo	RYGB-12_mo	NW
*n*	10	7	9	10
Gender F/M	5/5	–	–	7/3
Median age	50 ± 9	–	–	41 ± 15
Hypertension (+/total)	7/9	4/7	3/9	0
Diabetes (+/total)	7/9	1/7	1/9	0
Hyperlipidemia (+/total)	8/10	2/7	1/9	0
Arthritis (+/total)	9/10	7/7	9/9	0
Sample collection	N/A	216 ± 41 days after surgery	455 ± 124 days after surgery	N/A

Median and median absolute deviation values were reported for sample collection times.

Figure 1b, c shows the short- and longer-term effects of RYGB surgery on weight loss. Percent excess weight loss (%EWL) calculations, also shown in Fig. 1c, confirm that the participants achieved the greatest weight loss during the initial 6 months and maintained the weight loss a year after the surgery. Median %EWL after 12 months (65 ± 10) was slightly lower than the median %EWL of the cross-sectional group (RYGB-CS) (73 ± 15)^6^; however, this difference was not statistically significant (*p* = 0.24).

Changes in participants’ diet regimens may have contributed to weight loss after the surgery. It is important to note that dietary intake survey was self-administered; hence, errors in completion could have occurred. Table 2 summarizes total dietary calories and dietary composition. Based on total calories reported, the morbidly obese participants (pre-RYGB) were consuming fewer calories than the normal weight (NW) participants. In the United States, it is often recommended to have morbidly obese patients lose 10% of their excess weight prior to surgery in order to minimize surgical complications^1^, even though pre-surgical weight loss has not been associated with a reduction in post-operative complications^28^. Our pre-RYGB participants were enrolled in a pre-surgery diet program, and according to the self-reported surveys, they appear to have restricted their calorie intake to achieve pre-surgical weight loss. Although the caloric intake increased by 22% at 12 months compared to 6 months after RYGB, the weight loss benefits were sustained. The dietary composition of the morbidly obese participants did not significantly change after the surgery (Wilcoxon signed-rank test, *p* = 0.683), although, compared to NW individuals, carbohydrates formed a smaller fraction of the diets of post-RYGB participants (Table 2). Our study results are consistent with prior reports that RYGB results in significant weight loss, especially during the first six months after the surgery^29^, and remain stable or continue to improve until up to one year after the surgery^30^.

**Table 2:** Dietary composition of the samples of normal weight (NW), pre-surgical morbidly obese baseline (pre-RYGB), 6 months after surgery (RYGB-6_mo), and 12 months after surgery (RYGB-12_mo).

	NW (*n* = 10)	Pre-RYGB (*n* = 9)	RYGB- 6_mo (*n* = 7)	RYGB- 12_mo (*n* = 9)
Calorie intake (cal)	2160 ± 680	1820 ± 710	1310 ± 510	1420 ± 465
Carbohydrate %	50 ± 7	42 ± 8	40 ± 6	37 ± 6
Fat%	33 ± 6	36 ± 6	36 ± 5	38 ± 6
Protein %	14 ± 3	19 ± 4	20 ± 8	21 ± 8
Fiber intake (g)	21 ± 9	14 ± 12	18 ± 6	14 ± 5
Stool consistency/Bristol Stool Scale	NA	4 ± 1.2	4 ± 1.0	4 ± 1.1

Median values were presented with median absolute deviation values.

Besides weight loss, RYGB is known to lead to resolution of many metabolic disorders, including Type II diabetes. At their baseline measurements, seven participants had high blood pressure and diabetes, eight of them had hyperlipidemia, and nine of them had degenerative osteoarthritis. After RYGB, a majority of the study participants had resolution of diabetes, hyperlipidemia, and hypertension (Table 1), but not arthritis. The metabolic improvements after RYGB are well known and our observations are in agreement with previous reports^31,32^.

### RYGB altered fecal and mucosal microbiome structures

**Fig. 2:**
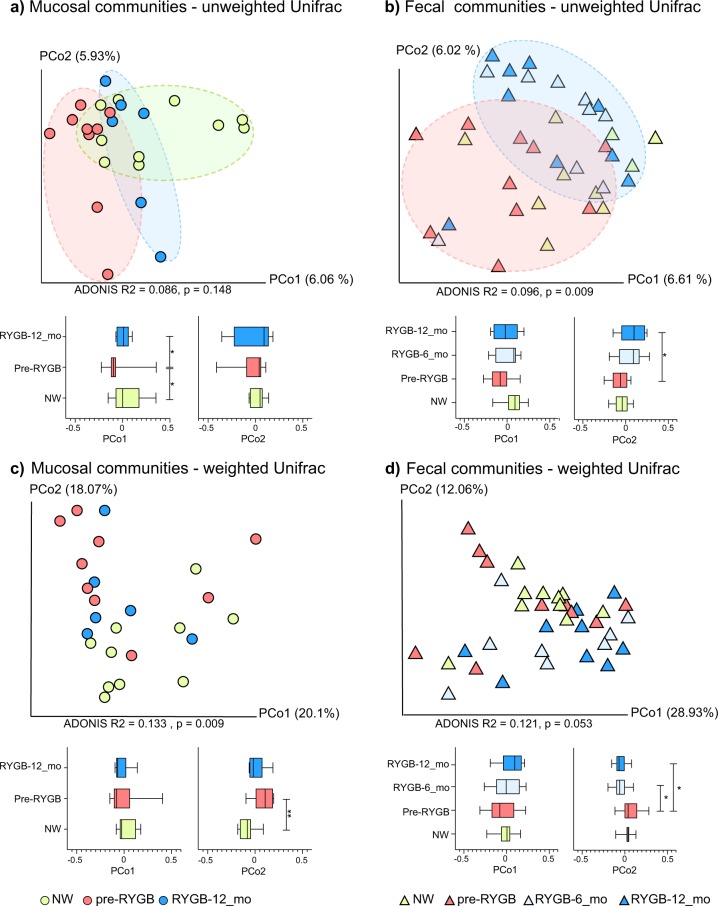
Unifrac analysis of mucosal and fecal microbiome after RYGB surgery. Microbiome communities (**a** mucosal) and (**b** fecal) before and after RYGB surgery in comparison to NW controls based on unweighted Unifrac distances. Microbiome communities (**c** mucosal) and (**d** fecal) before and after RYGB surgery in comparison to NW controls based on weighted Unifrac distances. Box plots represent the median distances among the communities on PCo1 and PCo2. * indicates Mann–Whitney *U*-test *p* < 0.05 and ** indicates Mann–Whitney *U*-test *p* < 0.01.

To detect changes in the gut microbiome after RYGB surgery, we analyzed the structure of fecal and mucosa-associated (mucosal) microbiomes of morbidly obese individuals (*n* = 9) before and after surgery. Rectal mucosal samples were collected at baseline and 12 months after RYGB via unsedated flexible sigmoidoscopy. Microbial DNA was extracted from fecal and mucosal samples. We performed weighted and unweighted Unifrac^33^ analyses on 16S rRNA gene sequences using the QIIME 1.9 suite^34^, and the principal component analyses (PCoA) are shown in Fig. 2. The effects of RYGB on the microbiome were pronounced for mucosal and fecal communities (Fig. 2a, b) for PCoA analysis based on unweighted Unifrac distances. As demonstrated by Fig. 2a, mucosal communities differed significantly on the PCo1 axis when comparing pre-RYGB group to RYGB-12_mo group (*p* = 0.02). Additionally, the pre-RYGB group was significantly different than the NW group, particularly on the PCo1 axis, indicating that microbiomes of normal weight and morbidly obese individuals differed in structure. Although PCoA based on unweighted Unifrac distances demonstrated the impact of RYGB on mucosal and luminal communities, PCo1 and PCo2 explained only a fraction (up to 13%) of the variability in the data set. Even though we controlled for factors such as, age of the participants and use of pharmaceuticals, heterogeneity in the human population, and other factors that influence gut microbiota composition led to a small fraction of variability in the data set being explained by the PCo1 and PCo2.

Differences in mucosal communities before and after RYGB were less apparent when weighted Unifrac (Fig. 2c) was used to calculate the dissimilarities among the communities. With weighted Unifrac analysis, the differences for minor taxa were obscured by the great abundances of Firmicutes and Bacteroidetes phylotypes.

Based on unweighted and weighted Unifrac distances, changes in the fecal microbiome appeared as soon as 6 months after the surgery, and the difference on PCo2 between pre-RYGB and RYGB-12_mo was significant (*p* = 0.04) (Fig. 2b, d). The ADONIS test was used on Unifrac distance matrices to differentiate overall differences in microbiome structure based on defined groups. The ADONIS *R*^2^ values ranged from 0.086 to 0.133 (*p* < 0.05) based on participant groups (NW, Pre-RYGB, RYGB-6_mo, and RYGB-12_mo) (Fig. 2). Even though these values are relatively small in terms of explaining the variation in the data set, ADONIS *R*^2^ values were smaller than 0.03 when grouping was based on gender, diet, BMI, stool consistency, or age. The ADONIS *R*^2^ results illustrate that our data set had high heterogeneity and variability; nevertheless, bariatric surgery had significantly greater impact on the overall microbiome structure than any of the other factors that commonly explain interpersonal variability, including diet and BMI.

When the RYGB-CS samples were incorporated into the weighted and unweighted Unifrac analysis, both RYGB-6_mo and RYGB-12_mo samples clustered together with RYGB-CS samples for weighted and unweighted Unifrac, although more strongly with the unweighted Unifrac (Supplementary Fig. 1, ADONIS *R*^2^ = 0.2401, *p* = 0.003). Additionally, when fecal and mucosal samples were analyzed together (Supplementary Fig. 1), clustering based primarily on sample type followed by the participant groups was observed, especially based on unweighted Unifrac distances. Interestingly, some of the RYGB-12_mo mucosal samples clustered with the fecal samples, indicating that after RYGB the mucosal community structure was more similar to the fecal community structure; however, the small sample size did not allow us to assess the significance of this observation. In summary, the results for the fecal microbiome are consistent with previous reports^4,7,10,12^ showing that fecal microbiome structure changed after RYGB, with changes sustained at least 1 year after surgery.

It is imperative to characterize changes in the microbiome of the mucosal space and the feces due to their differences and physiological relevance. In the lumen, substrates are usually dietary molecules, whereas in mucosal surfaces, they are host-derived glycans^35^. Another difference is the electron acceptor at the mucosal surfaces versus the lumen^36^. Oxygen derived from the eukaryotic tissues is gradually depleted in the mucosal layer by facultative anaerobes, and, therefore, the lumen becomes anaerobic^18^. Microorganisms that live in the lumen are also affected by other host-associated factors such as transit time, frequency and composition of dietary intake, and bile acids^37^.

### Fecal microbiome after RYGB was similar to microbiome from a RYGB-CS cohort

**Fig. 3:**
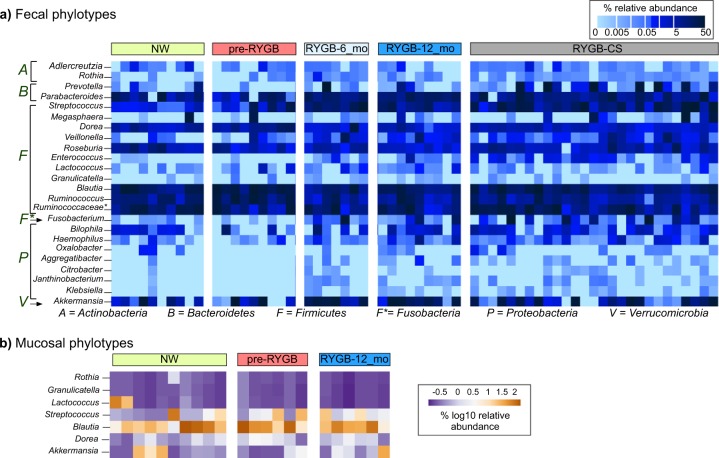
Relative abundance of genus-level phylotypes after RYGB surgery. Heat map showing which **a** fecal and **b** mucosal microbial phylotypes were enriched or depleted after RYGB surgery. The samples were chronologically ordered based on time after surgery. The statistical significance between pre-RYGB and RYGB-12_mo groups were based on Wilcoxon signed-rank test and *p* values were corrected using Bonferroni method *p < 0.05.

Figure 3a shows the relative abundances of significantly enriched or depleted genus-level phylotypes in the lumen 6 months and 1 year after RYGB surgery and in comparison to a cross-sectional cohort (RYGB-CS). The surgery significantly altered relative abundances of 24 genus-level phylotypes (Wilcoxon signed-rank test *p* < 0.05). The majority of enrichments or depletions of genus-level phylotypes occurred within the first 6 months after surgery and were sustained 1 year after surgery (Fig. 3a and Supplementary Fig. 2). The abundances of these phylotypes were significantly different in RYGB-6_mo and RYGB-12_mo groups, compared to the NW group (Supplementary Fig. 2) (Mann–Whitney *U*-test *p* < 0.05). One of the microbial staples of RYGB surgery is enrichment of phylotypes from *Gammaproteobacteria*^4,7^, and our analysis showed that RYGB also altered the abundance of genus-level phylotypes from other phyla, including *Firmicutes*, Actinobacteria, Fusobacteria, and Bacteroidetes.

We observed an increase in the abundance of Proteobacteria phylotypes *Rothia*, *Aggregatibacter*, *Granulicatella, Citrobacter*, *Janthinobacterium*, and *Klebsiella*. Firmicutes had many phylotypes whose relative abundances were affected by the surgery. While many phylotypes—such as *Streptococcus*, *Enterococcus*, *Lactococcus*, *Veillonella*, and *Granulicatella*—were enriched, *Ruminococcus*, *Blautia*, and *Roseburia* were depleted after the surgery. *Akkermansia* from Verrucomicrobia and *Adlercruetzia* and *Rothia* from Actinobacteria also were in greater abundance after RYGB.

Figure 3a also shows the relative abundance of the aforementioned phylotypes compared to a RYGB-CS group. The RYGB-CS group consisted of participants who had previously undergone RYGB, had lost at least 50% of their excess weight, and who had provided a stool sample 13–60 months after surgery; therefore, it was a more heterogeneous group by time after surgery^6^. The results seen in the RYGB-CS group paralleled those seen in the RYGB-6_mo and RYGB-12_mo groups (Supplementary Fig. 2). The results of the RYGB-CS group were sorted based on the time after their surgery, and we found no clustering based on time after surgery. These results support that changes in the microbiome occurred quickly after the surgery (within 6 months) and persisted in the long term (>60 months).

To confirm observations on genus-level phylotypes, we performed unsupervised clustering and generated hierarchical clustering heat map based on Euclidean distances among the samples (Supplementary Fig 3). Samples formed five distinct clusters driven by *Fusobacterium, Prevotella*, *Ruminococcus*, *Parabacteroides*, *Blautia*, and *Akkermansia*. Three of the clusters were composed of only post-RYGB samples (RYGB-6_mo, RYGB_12-mo, and RYGB-CS), indicating the impact of the RYGB alone on the relative abundance of genus-level phylotypes. The sustained changes observed in the microbiome after RYGB indicate that the surgery-imposed changes to the gut environment/ecosystem were persistent and permanently affected gut microbiota in a stronger way than interpersonal variations.

### RYGB altered mucosal microbial communities increasing *Akkermansia* sp. and lactate metabolizers

Our analysis of enriched or depleted phylotypes also demonstrates that RYGB surgery led to a wide spectrum of changes in the mucosal space (Fig. 3b). Six genus-level phylotypes were significantly enriched in the mucosa after RYGB surgery: *Granulicatella*, *Lactococcus*, *Streptococcus*, *Blautia*, *Dorea*, and *Akkermansia* (Supplementary Fig. 4) (Wilcoxon signed-rank test *p* < 0.05). Relative abundances of these phylotypes also were greater in the NW mucosa, compared to the pre-RYGB mucosa. Except for *Akkermansia*, the microorganisms enriched post-surgery are from the Firmicutes phylum and are known to form biofilms and contribute to lactate metabolism^38^. *Lactococcus*, *Streptococc*us, and *Granulicatella* are lactate-producing microorganisms, whereas *Dorea* and *Blautia* are lactate oxidizers^39^. Lactate-producing *Streptococcus* and *Lactococcus* species have been used as probiotics to enhance gut epithelial barrier and integrity^40^, since lactate availability is crucial for butyrate producers and, therefore, colon epithelium health^41^.

In addition to lactate producers and oxidizers, we observed an increase in the relative abundance of *Akkermansia* in the mucosa after RYGB surgery. Previously, a similar trend was observed in mice after RYGB^22^, and our findings confirmed this observation in humans. Animal models have demonstrated that a weak gut barrier contributes to the development of endotoxemia and inflammation, which subsequently leads to insulin resistance and an increase in adiposity^42–44^. *Akkermansia* is a known mucin degrader, and its presence has been shown to improve the gut epithelial barrier, reduce adiposity of the organs, and protect against insulin resistance and obesity in humans^45^. However, a recent study that investigated the link between *Akkermansia* abundance in the feces of severely obese individuals after RYGB and diabetes did not report any association between *Akkermansia* abundance and glucose homeostasis after RYGB^46^. Overall, our results indicate that alterations in the gastrointestinal mucosa after RYGB may contribute to an increase in mucin-degrading, lactate-producing, and lactate-oxidizing microorganisms.

### Post-RYGB microbiota alters the fecal metabolome

**Fig. 4:**
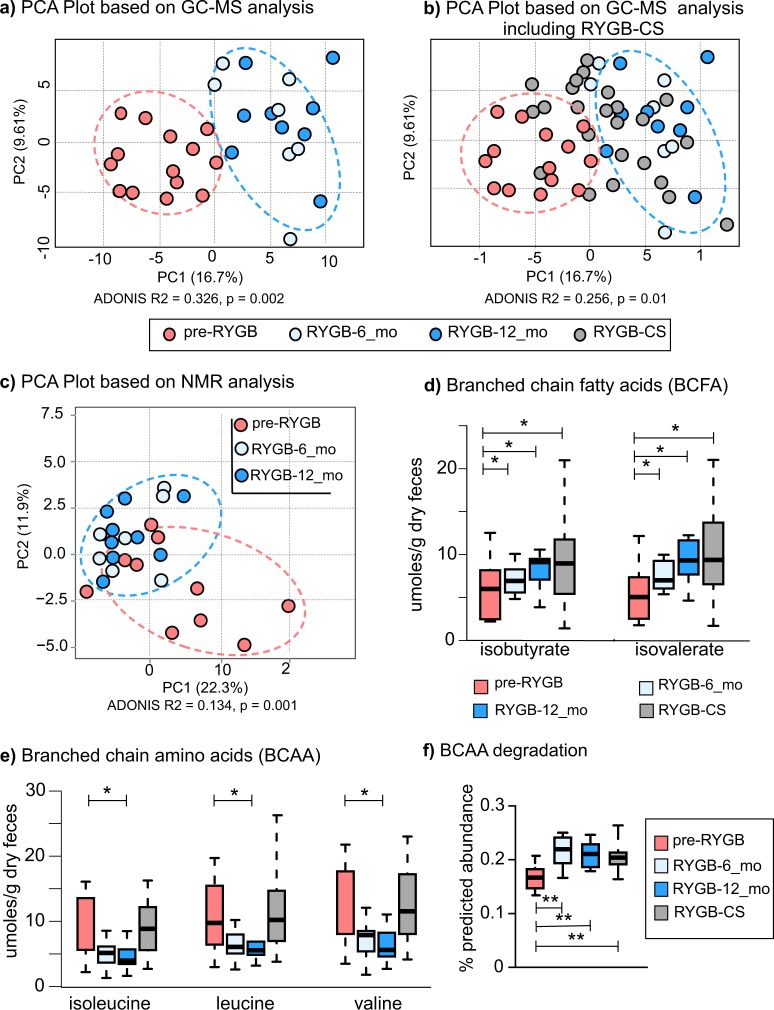
Fecal metabolites detected with H-NMR and GC-MS. Principal component analysis based on metabolites detected by **a** GC-MS based metabolomes before and after the surgery. **b** Principal component analysis based on GC-MS metabolome that includes RYGB-CS group samples. **c** ^1^H-NMR based metabolomes before and after the surgery. **d** Branched-chain fatty acids—isobutyrate and isovalerate—measured with NMR after RYGB surgery prospectively and retrospectively. **e** Concentrations of isoleucine, leucine, and valine, branched-chain amino acids (BCAA) measured with NMR after RYGB surgery prospectively and retrospectively. The box plots represent minimum, maximum, median, first quartile and third quartile values. **f** Predicted relative abundance of the genes that are involved in branched chain amino acid (leucine, valine, and isoleucine) degradation. KEGG ko00280 valine, leucine, isoleucine degradation pathway was used in the analysis. * indicates statistical significance between pre-RYGB and RYGB-12_mo groups based on Wilcoxon signed-rank test and *p* values were corrected using Bonferroni method *p < 0.05.

Changes in the structure of the gut microbiome after RYGB surgery were reflected in the gut metabolome. Figure 4 shows PCA results for the fecal metabolomes detected by gas chromatography-mass spectrometry (GC-MS) and ^1^H-NMR-based methods. Fecal water-soluble extracts were analyzed with ^1^H-NMR, while lyophilized fecal matter was analyzed with GC-MS. ^1^H-NMR provided mainly volatile and water-soluble compounds, whereas GC-MS identified many metabolites of the undigested nutrients and components of microbial cells.

Based on GC-MS data (Fig. 4a), RYGB-6_mo and RYGB-12_mo fecal metabolomes clustered away from pre-RYGB metabolomes (ADONIS *R*^2^ = 0.326, *p* = 0.002). Figure 4b overlays the GC-MS-based metabolomes of the RYGB-CS participants to the metabolomes of longitudinal study participants illustrated in Fig. 4a. The metabolomes of most of the RYGB-CS participants were more similar to RYGB-6_mo and RYGB-12_mo participants (Mann–Whitney *U*-test *p* < 0.05, ADONIS *R*^2^ = 0.256, *p* = 0.01) than to Pre-RYGB or NW participants, supporting the observation that the impact of RYGB surgery resulted in a unique metabolic fingerprint that was preserved in the long term. Moreover, the similar clustering patterns with the metabolome (Fig. 4a, b) and the microbiome (Fig. 2a) strengthen the conclusion that the changes in the microbiome and metabolome after RYGB surgery were linked, persistent, and stronger than interpersonal variations.

Similar to GC-MS metabolome, ^1^H-NMR quantification of water-soluble metabolites showed a distinct RYGB metabolome (Fig. 4c). The concentrations as measured by ^1^H-NMR of the major SCFAs of the human gut—acetate, butyrate, and propionate (Table 3)—were similar for the RYGB-CS and RYGB-12_mo groups. Propionate-to-acetate and butyrate-to-acetate ratios increased 6 and 12 months after the surgery, and the difference between baseline and 12-month samples was statistically significant (*p* = 0.03). We previously reported a similar trend with our RYGB-CS cohort^6^. Higher butyrate- and propionate-to-acetate ratios after the surgery compared to baseline indicates a shift in microbial metabolism from acetate production to butyrate and propionate production. Butyrate and propionate have been shown to signal free fatty acid receptors and induce a satiety response in the brain of mice^47^. Shifts in microbial metabolism reflect another potential mechanism explaining how microorganisms contribute to weight loss following RYGB.

**Table 3:** Concentrations of acetate, butyrate, and propionate normalized to dry weight g stool, along with propionate-to-acetate and butyrate-to-acetate ratios in NW, pre-RYGB, RYGB-6_mo, and RYGB-12_mo, and RYGB-CS groups.

	Propionate/acetate	Butyrate/acetate	μmoles/g stool		
			Acetate	Butyrate	Propionate
NW	0.40	0.25	110 ± 46	31 ± 16	45 ± 17
Pre-RYGB	0.37	0.24	240 ± 109	47 ± 11	75 ± 20
RYGB-6_mo	0.38	0.27	240 ± 56	61 ± 20	100 ± 16
RYGB-12_mo	0.41	0.30	160 ± 21	42 ± 9	70 ± 9
RYGB-CS	0.41	0.34	180 ± 25	69 ± 16	81 ± 12

The measurements were taken with ^1^H-NMR. The numbers represent median values of the groups with median absolute deviation values. Wilcoxon signed-rank test was used to assess statistical significance. Propionate/acetate and butyrate/acetate ratios were significantly different between the Pre-RYGB and RYGB-12_mo groups (Wilcoxon rank-signed test *p* = 0.004 and p = 0.002).

We also evaluated the concentrations of branched chain amino acids (BCAA) and their fermentation products—branched-chain fatty acids (BCFA)—before and after RYGB. As seen in Fig. 4d, the fecal concentrations of two BCFAs—isobutyrate and isovalerate—increased after surgery and this observation is consistent with previous observations^8,13^. The RYGB-CS and RYGB-12_mo groups had similar concentrations of these BCFAs. Therefore, we can deduce that an increase in the abundance of these BCFAs was more likely associated with RYGB. Three BCAAs—leucine, isoleucine, and valine—were at significantly lower abundance in RYGB-12_mo in comparison to NW and RYGB-CS groups (Fig. 4e, Table S1). Interestingly, the concentration of BCAAs poorly correlated with the amount of protein consumed by the participants (Spearman’s rank correlation coefficient < 0.335). Even though BCAA concentrations were variable, their fermentation products were always greater post-RYGB. This observation was further supported by the predicted abundances of the genes that are involved in the BCAA (valine, leucine, and isoleucine) degradation and the synthesis of BCFA production pathways as shown in Fig. 4f. The predicted abundances of BCAA degradation genes were significantly greater after RYGB and in the RYGB-CS group in comparison to the pre-RYGB group. In summary, changes in the microbiome due to RYGB surgery seemed to enhance fecal amino acid metabolism, which may have contributed to weight loss by producing BCFA that are capable of signaling free fatty acid receptors^48^. The role of BCFAs on FFA receptor signaling warrants further investigation.

In addition to SCFAs and BCFAs, we analyzed a wide spectrum of other metabolites. Most of the fecal metabolites including sugars and amino acids, detected with ^1^H-NMR and GC-MS, were at greater abundance in the pre-RYGB group, and their concentrations dropped 12 months after surgery (Table 4). The fecal metabolite concentration profiles of RYGB-12_mo and RYGB-CS groups were similar, possibly due to the altered gastrointestinal tract environment after the surgery and similarities in participant diets. However, as shown in Table 4, besides isovalerate and isobutyrate, concentrations of xylose also increased after RYGB and were even higher than for NW controls. Greater abundance of fecal xylose after RYGB would seem to indicate that the participants adapted to more plant-based diets or that they lost some microbial hydrolytic capabilities to metabolize xylose. The (self-reported) participants’ fiber intake did not change significantly after the surgery (Table 1), although it was statistically lower in post-RYGB participants compared to NW participants (*p* = 0.032).

**Table 4:** Concentrations of fecal metabolites normalized to dry weight that were statistically different between pre-RYGB and RYGB-12_mo samples.

µmoles/g stool dry weight					*p* value
Metabolites	NW	Pre-RYGB	RYGB-6_mo	RYGB-12_mo	Pre-RYGB vs RYGB-12_mo
Alanine	10.0 ± 4.3	11.8 ± 5.0	9.7 ± 2.0	6.9 ± 1.3	0.035
Cadaverine	0.2 ± 0.0	0.4 ± 0.4	0.6 ± 0.2	0.7 ± 0.3	0.031
Glucose	5.8 ± 4.1	26.1 ± 11.1	6.1 ± 4.2	3.9 ± 1.4	0.002
Glutamine	3.5 ± 1.7	5.8 ± 2.0	2.6 ± 1.6	2.5 ± 1.0	0.051
Isopropanol	0.2 ± 0.1	0.1 ± 0.0	0.2 ± 0.0	0.2 ± 0.0	0.036
Methanol	0.9 ± 0.5	1.7 ± 0.7	1.1 ± 0.6	0.7 ± 0.1	0.006
Succinate	2.9 ± 0.5	4.6 ± 0.5	2.5 ± 0.1	2.1 ± 0.3	0.028
Taurine	0.7 ± 0.0	8.0 ± 7.9	0.0 ± 0.0	0.0 ± 0.0	0.003
Threonine	5.4 ± 2.2	9.4 ± 3.7	4.7 ± 2.6	3.9 ± 1.2	0.035
Thymidine	0.2 ± 0.0	0.8 ± 0.7	0.0 ± 0.0	0.0 ± 0.0	0.009
Tyrosine	5.0 ± 2.0	7.8 ± 2.8	4.8 ± 1.4	3.4 ± 0.7	0.042
Uracil	2.4 ± 1.2	3.4 ± 1.4	1.9 ± 0.8	2.1 ± 0.5	0.035
Uridine	0.0 ± 0.0	1.3 ± 1.3	0.0 ± 0.0	0.0 ± 0.0	0.019
Valerate	7.9 ± 1.1	11.9 ± 3.1	0.0 ± 0.0	0.0 ± 0.0	0.06
Valine	7.4 ± 3.5	11.9 ± 5.2	7.8 ± 2.1	5.6 ± 1.3	0.035
Xylose	1.2 ± 1.0	3.1 ± 1.5	0.0 ± 0.0	0.0 ± 0.0	0.000
Leucine	6.6 ± 3.1	9.7 ± 4.3	6.1 ± 1.4	5.5 ± 1.1	0.042
Lysine	4.4 ± 1.2	7.4 ± 3.6	3.8 ± 1.7	2.5 ± 1.0	0.035
Isoleucine	5.4 ± 2.4	9.0 ± 4.2	5.1 ± 1.4	4.0 ± 1.1	0.028
Isovalerate	5.0 ± 2.4	5.0 ± 2.4	7.0 ± 1.6	9.3 ± 2.3	0.023
Isobutyrate	4.8 ± 1.9	6.0 ± 2.6	6.9 ± 1.6	9.1 ± 0.8	0.018

The measurements were done with ^1^H-NMR. The numbers represent median values of the groups with median absolute deviation values. Wilcoxon signed-rank test was used to assess statistical significance.

### RYGB surgery decreased fecal bile acid concentrations

**Fig. 5:**
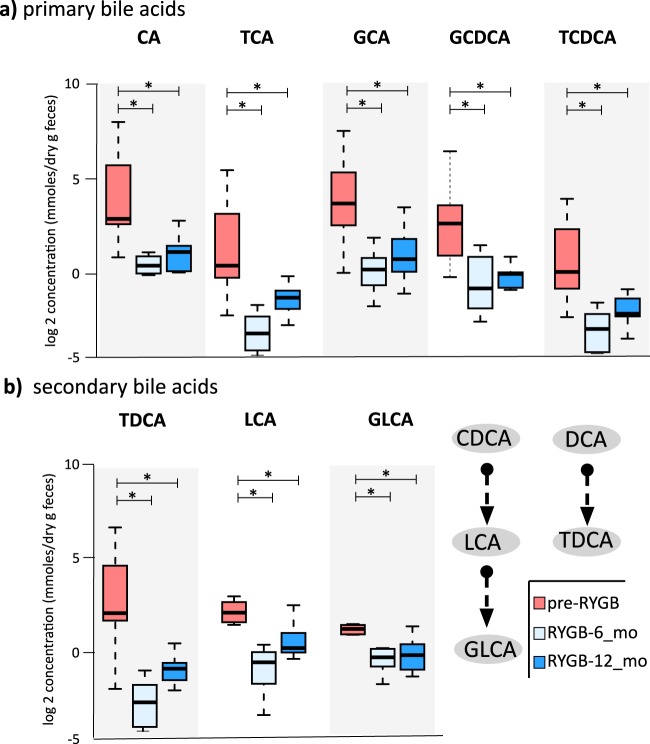
Fecal bile acids measured before and after RYGB surgery. **a** Fecal primary bile acid (CA cholic acid, TCA taurodeoxycholic acids, GCA glycocholic acid, GCDCA glycochenodeoxycholic acid, TCDCA taurochenodeoxycholic acid) and **b** fecal secondary bile acids (TDCA taurodeoxycholic acid, LCA lithocholic acid, GLCA glycolithocholic acid) that were statistically different after RYGB surgery. * indicates statistical significance between pre-RYGB and RYGB-6 and RYGB-12_mo groups based on Wilcoxon signed-rank test and *p* values were corrected using Bonferroni method *p < 0.05. The box plots represent minimum, maximum, median, first quartile, and third quartile values.

**Fig. 6:**
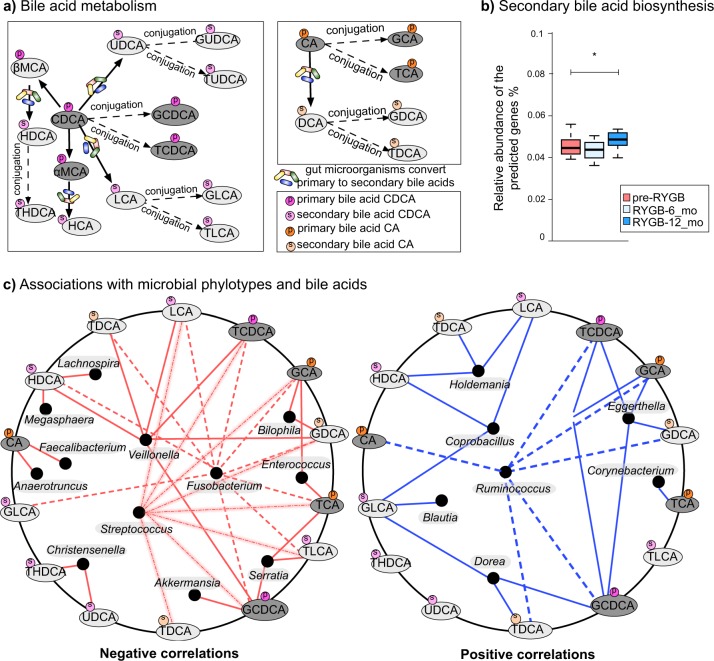
Bile acid–fecal microbiota interactions. **a** Bile acid transformation reactions. Orange color = primary bile acids, green = primary conjugated bile acids, blue = secondary bile acids, pink = secondary conjugated bile acids. CA cholic acid, DCA deoxycholic acid, GDCA glycodeoxycholic acid, TDCA taurodeoxycholic acid, GCA glycocholic acid, TCA taurocholic acid, CDCA chenodeoxycholic acid, LCA lithocholic acid, HCA hyacholic acid, HDCA hyodeoxycholic acid, UDCA ursodeoxycholic acid, GUDCA glycoursodeoxycholic acid, TUDCA tauroursodeoxycholic acid, THDCA taurohydroxydeoxycholic acid, GLCA glycolithocholic acid, TLCA taurolithocholic acid, GCDCA glycochenodeoxycholic acid, TCDCA taurochenodeoxycholic acid, αMCA α-muricholic acid, βMCA β-muricholic acid. **b** Bile acid biosynthesis genes predicted from 16S rRNA gene abundances via PICRUSt. KO numbers that were used in the prediction: K01442, K00076, K23231, K22604, K22605, K22606, K22607, K15868, K15871, K15869, K15870, K15873, K15874, and K07007. The box plots represent minimum, maximum, median, first quartile, and third quartile values. **c** Fecal bile acid and microbiome co-occurrence network based on Spearman’s rho correlation coefficients.

As RYGB is known to alter the bile acid metabolism^49,50^ and contribute to remission of type 2 diabetes and weight loss^51^, we quantified seven primary and 10 secondary bile acids in fecal samples from participants before and after RYGB surgery using liquid chromatography-mass spectrometry (LC-MS). Figure 5a, b and Table S2 show primary and secondary fecal bile acids and their conjugated forms measured at baseline, 6 months, and 12 months after RYGB. Fecal concentrations of primary bile acid—cholic acid (CA)—and its glycine- and taurine-conjugated forms (TCA and GCA) were significantly lower 6 months after the surgery (CA *p* = 0.022, TCA *p* = 0.001, GCA *p* = 0.002), and they remained at similar concentrations 12 months after surgery. Similarly, concentrations of glycine- and taurine-conjugated forms of chenodeoxycholic acid (CDCA), GCDCA and TCDCA, dropped significantly 6 months after the surgery. Concentrations of secondary bile acids, lithocholic acid (LCA), its glycine conjugated form, GLCA, and taurodeoxycholic acid (TDCA) significantly dropped 6 months after surgery as well (LCA *p* = 0.02, GLCA *p* = 0.001, and TDCA *p* = 0.003). Figure 6a illustrates the conjugation and transformation reactions of primary bile acids and the resulting secondary bile acids produced by gut microbiota^52^. Our findings show that primary and secondary bile acids were significantly diminished in feces after RYGB surgery.

In order to reveal microbial connections to bile acid metabolism, we used Phylogenetic Investigation of Communities by Reconstruction of Unobserved States (PICRUSt) software^53^ to predict secondary bile acid biosynthesis pathway from 16S rRNA gene abundances. PICRUSt prediction of secondary bile acid biosynthesis pathway was greater after RYGB; however, these are genomic predictions and not activity measurements (Fig. 6b). Table S2 summarizes the median concentrations of primary and secondary bile acids observed in NW and RYGB-CS groups in comparison to RYGB-12_mo and pre-RYGB groups. The concentrations measured in the RYGB-CS group were similar to the RYGB-12_mo group, which indicates that the response of surgical modification on bile acid metabolism was strong and reproducible, even if the baseline time points before the surgery are missing. Additionally, bile acid levels after RYGB groups were similar to NW participants (Table S2). Overall, our findings indicate that fecal concentrations of primary and secondary bile acids declined after RYGB surgery, and levels similar to those in NW individuals were maintained even years after the surgery. Fat and cholesterol intake are important factors in the production and secretion of bile acids^52^. As seen in Table 1, the participants did not reduce the fat percentage of their diets, although they consumed fewer calories after RYGB, which leads to lower absolute amounts of fat being consumed. Lower delivery of fat to the gastrointestinal tract might have played a role in the lower concentrations of fecal primary and secondary bile acids measured in this study.

Considering that gut microbiota can transform bile acids^52,54^ and concentrations of bile acids can affect gut microbiota composition^52^, we performed co-occurrence-network analysis between fecal genus-level microbial phylotypes and bile acids. As shown in Fig. 6c, phylotypes that were enriched after RYGB, including *Fusobacterium*, *Veillonella*, *Enterococcus*, *Akkermansia*, and *Streptococcus* negatively correlated with various bile acids such as TDCA, LCA, TCDCA, GCA, GDCA, TCA, and TLCA. *Christensenella*, a strongly heritable phylotype that was also associated with lean body type^55^, was the only genus-level phylotype that negatively correlated with the secondary bile acids THDCA and UDCA. Previously, UDCA treatment have been associated with weight gain in humans^56^. On the other hand, *Ruminococcus*, *Coprobacillus*, *Holdemania*, *Eggerthella*, and *Dorea* positively correlated with primary and secondary bile acids.

We performed the same analysis with mucosal genus-level phylotypes and bile acids (see Supplementary Fig. 5). We observed associations with minor taxa such as *Methanobacterium* and bile acids. Interestingly, *Clostridium* genus phylotypes negatively correlated with a number of bile acids. Additionally, UDCA, GDCA, and GUDCA were the bile acids that showed the greatest number of associations with mucosal phylotypes. Given that bile acids have been reported to modify the gut microbiome^52^, lower delivery of bile acids to the colon might have played a role on some of the microbiome compositional changes observed. Additionally, microbial bile acid metabolism can potentially have effects on host body weight and metabolism since it was previously shown that bile diversion to the small intestine can recapitulate some of metabolic benefits of the RYGB independently from the surgery^57^.

Previous studies in humans reported increased levels of circulating bile acids, especially secondary bile acids, after RYGB as measured in blood plasma^25,51,58^. A recent study characterizing bile acids in the fecal samples in women after RYGB showed decreased concentrations of many bile acids^59^; hence, our results support findings from that study. In rats, RYGB has also been shown to increase plasma bile acid concentrations and the secretion of weight-loss-associated hormones Peptide YY and Glucagon Like Peptide-1 (ref. ^49^). However, a recent study on rats demonstrated that the bile acid profiles in the intestines did not change after RYGB even though microbial profiles were significantly altered^60^. One difference among the reported human studies and ours is that our measurements were in fecal samples, whereas the others analyzed serum samples; hence, the measurements are not directly comparable. Bile acid quantification is often done in serum samples, which might reflect more physiologically relevant concentrations. However, our findings in fecal samples may lead to more profound understanding of microbial metabolism of bile acids in the gut. Further studies on the impact of microbial metabolism and gut levels of bile acids on host health are warranted.

We demonstrated the impact of RYGB surgery on the gut microbiome, metabolome, and bile acid metabolism of humans studied prospectively and retrospectively. We document that changes in the human gut microbiome after RYGB in the luminal and mucosal space. The mucosal space is a critically important site for host–microbe interactions. Changes in the fecal metabolome mirrored changes in the fecal and mucosal microbiome structure, suggesting that the profile of microbial metabolism changed as a result of major physiological, environmental, and nutritional alterations affecting the gut after RYGB surgery. The delivery of bile acids to the colon diminished after surgery, potentially contributing to the altered microbiome and metabolome profiles. As a small sample size is a limitation of our study, studies with greater sample size are needed to validate our findings. Finally, results from a longitudinal cohort were consistent with observations from cross-sectional studies after RYGB surgery, supporting a dominant and persistent impact of RYGB on the intestinal microbiome.

## Methods

### Study design

For the longitudinal cohort, we recruited 10 morbidly obese participants who were scheduled to undergo RYGB surgery (pre-RYGB) and 10 normal weight controls. The demographics of the study participants are included in Table 1. Considering that RYGB cohorts are often composed of female participants^61^, our study presents a more balanced distribution of genders (see Table 1). In order to confirm results of cross-sectional studies with this longitudinal study, we included 24 participants (RYGB-CS) who had undergone RYGB surgery 13–60 months before the sample collection and had lost at least 50% of their excess weight. Therefore, the CS population represents long-term outcomes of RYGB surgery on gut microbiome and metabolome. The demographics of this cross-sectional population can be found in a previous publication^6^. Fecal samples collected at the specified time points (Fig. 1a) were stored at −80 °C within 4 h of production until analyzed. Three participants did not provide fecal samples at 6 months and one did not provide a sample at 12 months. Distal sigmoid colon (25 cm from the anal verge) biopsies were collected during non-sedated flexible sigmoidoscopy following administration of a cleansing enema from 10 NW participants and 9 prospective RYGB participants before and 12 months after the surgery at Mayo Clinic, Scottsdale, Arizona, USA. The samples were instantly washed and submerged in liquid nitrogen until frozen and were kept at −80 °C until analysis. All participants filled out 4-day food diaries and food-frequency questionnaires (within 2 weeks prior to sample collection) with assistance of a dietitian and DietOrganizer software (dietorganizer.com) was used to analyze the dietary composition.

### DNA extraction, 16S rRNA gene sequencing, and analysis

We extracted microbial DNA from feces and biopsy (mucosal) samples using MOBIO PowerSoil DNA extraction kit (MOBIO Laboratories, Carlsbald, CA, USA). We prepared sequencing libraries using the protocols from Earth Microbiome project using V4 primers with Illumina Miseq Instrument^62^. PANDAseq^63^ paired reads were analyzed using QIIME 1.9 suite^34^. The details of the analysis can be found in the Supplementary Document. Briefly, OTUs were formed at 99% sequence similarity and the OTUs that contained less than 0.005% of the total number of sequences and chimeric sequences were omitted from the analysis as previously recommended^64^. We calculated alpha and beta diversity metrics of Phylogenetic Diversity Whole Tree^65^, and Unifrac^33^. Gene abundances for bile acid biosynthesis were predicted with Phylogenetic Investigation of Communities by Reconstruction of Unobserved Species (PICRUSt) software^53^. Genus-level phylotypes that significantly differed after RYGB were clustered based on Euclidean distances using ClustVis^66^.

### ^1^H-NMR analysis of water-soluble fecal metabolites

For each fecal specimen, approximately 1 g of wet weight was diluted with 20 mL of milliQ water and homogenized by vortexing for 3 min. The homogenates were centrifuged at 16,110 × *g* for 15 min, and the supernatants were filtered through 0.2-μm PVDF membranes (PALL Corporation). The fecal extracts were diluted with a 10% (v/v) spike of a National Institute of Standards and Technology calibrated reference solution. The resulting mixture was loaded into 3-mm NMR tubes (Bruker Inc), and NMR spectra were collected using a Varian Direct Drive 600 MHz NMR spectrometer equipped with a 5 mm triple-resonance salt-tolerant cold probe. The 1D ^1^H NMR spectra of all samples were processed, assigned, and analyzed by using Chenomx NMR Suite 8.1 with quantification of metabolites based on spectral intensities relative to the internal standard and as previously described^6^.

### LC-MS analysis of fecal bile acids

Fifty microliters of internal standard mixture (1.0 µg/mL) were spiked into 5 mg of lyophilized fecal samples and processed as described in the Supplementary document. Homogenized samples were centrifuged at 13,600 × *g* for 20 min and the supernatants were filtered using Acrodisc 45 µm syringe-filters. Samples were cleaned-up using a 60 mg Oasis HLB 3cc cartridge (Waters Corporation, Milford, MA), dried in vacuo, and stored at −70 °C until analysis. The extracts were analyzed with a Waters nano-Acquity UPLC system (Waters Corporation, Milford, MA). MS analysis was performed using an Agilent model 6490 triple quadrupole mass spectrometer (Agilent Technologies, Santa Clara, CA) outfitted with an in-house nano-electrospray ionization interface. The sample preparation and bile acid quantification procedures were based on the method of Humbert et al.^67^, with modifications described in the Supplementary Document.

### GC-MS analysis of fecal metabolites

Metabolites were extracted from 10 mg of lyophilized stool samples using methanol with sonication. Extracted metabolites were completely dried in vacuo and derivatized by methoxylamination and trimethylsilyation and analyzed by GC-MS as reported previously^68^. GC-MS raw data files were processed using the Metabolite Detector software, version 2.5 beta^69^. All raw GC-MS data will be made available via the MetaboLights metabolomics data repository (http://www.ebi.ac.uk/metabolights/index).

### Statistical analyses of microbiome and metabolome data sets

We used Statistical Package for Social Sciences (SPSS) and R packages^70^ for all statistical analyses. The medians of the groups along with median absolute deviation values were calculated and reported. Shapiro–Wilk test was used to test normality of the data sets. For the longitudinal cohort, 16S rRNA gene relative abundance comparisons were tested with Wilcoxon signed-rank test. For cross-sectional cohort comparisons, Mann–Whitney’s *U*-test was used. The *p* values were corrected using Benjamini and Hochberg method^71^ and corrected *p* values less than 0.05 were accepted as significant. Same tests were utilized to analyze NMR, GC-MS, and LC-MS data sets. For the LC-MS data analysis, the data were analyzed after they were log2 transformed. We performed ADONIS test^72^ on microbiome and metabolome distance matrices to quantify the variation explained by defined variables based on 999 permutations. To reveal associations between bile acid concentrations and the relative abundance of taxonomic groups, we calculated Spearman’s rank correlation coefficient and accepted significance above critical values with Bonferroni corrected *p* values less than 0.05.

### Ethics approval and consent to participate

All study participants provided written informed consent and all procedures were approved by the Institutional Review Boards of Mayo Clinic and Arizona State University (IRB# 10-008725).

### Reporting summary

Further information on research design is available in the Nature Research Reporting Summary linked to this article.

## Supplementary information

Supplementary Information

Reporting Summary

## Data Availability

The 16S rRNA gene sequences were deposited in the Sequence Read Archive (SRA) database (BioSample IDs = SAMN08684029-SAMN08684111).
