# Supplementary material for: Temporospatial shifts in the human gut microbiome and metabolome after gastric bypass surgery
Source: NPJ Biofilms Microbiomes. 2020 Mar 13;6:12. doi: 10.1038/s41522-020-0122-5 (PMC7070067; doi:10.1038/s41522-020-0122-5)
Supplement: Supplementary file 1 — Supplementary Information [file 41522_2020_122_MOESM1_ESM.pdf]

Supplementary Figures

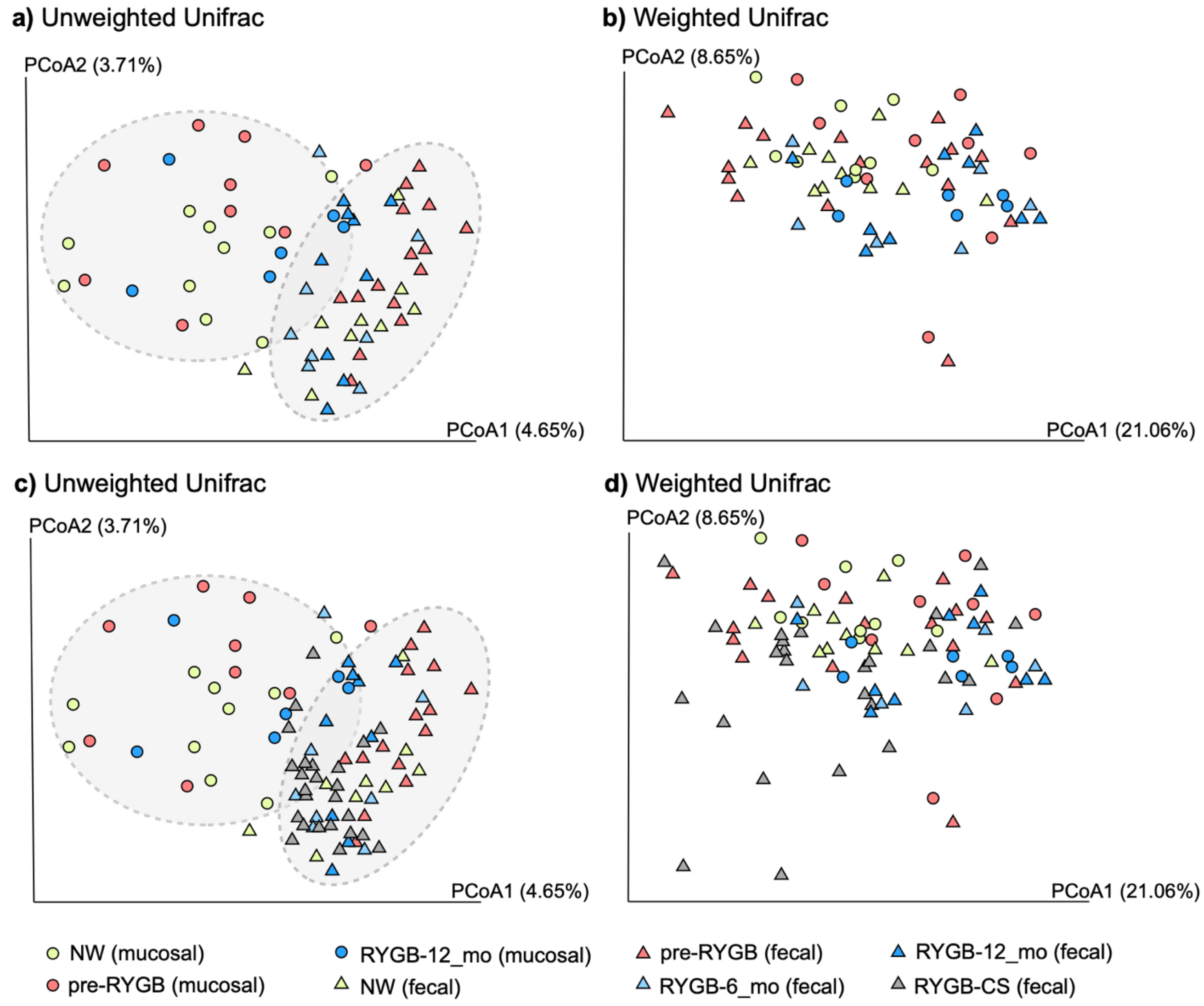

Supplementary Figure 1. Unifrac analysis of mucosal and fecal microbiomes analyzed together. **a.** Unweighted and **b.** weighted Unifrac distances shown on principal component analysis plots for biopsy and fecal samples from the prospective cohort. **c.** Unweighted and **d.** weighted Unifrac distances shown on principal component analysis plots that include RYGB-CS samples. Clustering was more apparent with unweighted Unifrac analysis.

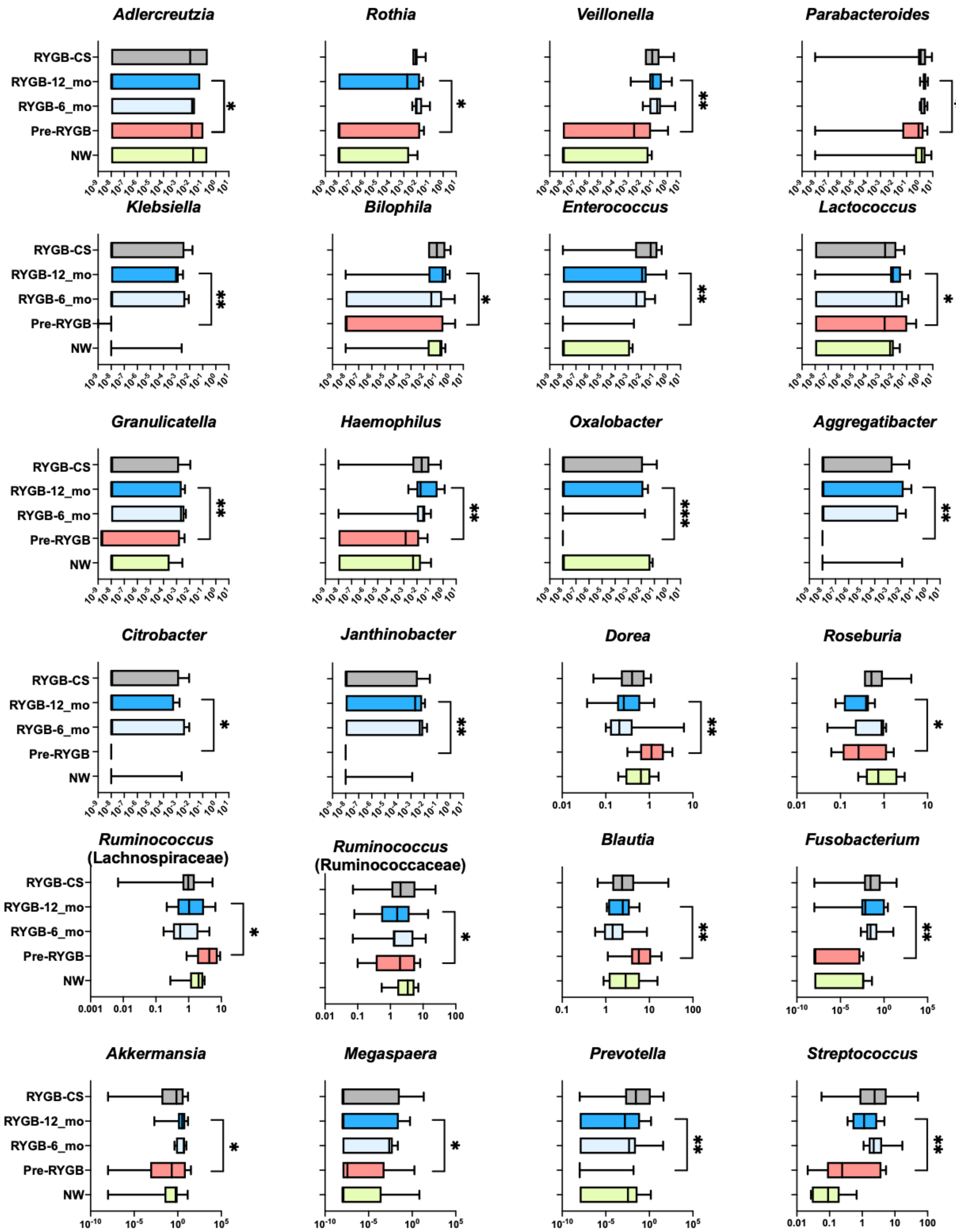
Adlercreutzia
Rothia
Veillonella
Parabacteroides
Klebsiella
Bilophila
Enterococcus
Lactococcus
Granulicatella
Haemophilus
Oxalobacter
Aggregatibacter
Citrobacter
Janthinobacter
Dorea
Roseburia
Ruminococcus (Lachnospiraceae)
Ruminococcus (Ruminococcaceae)
Blautia
Fusobacterium
Akkermansia
Megaspaera
Prevotella
Streptococcus
RYGB-CS
RYGB-12_mo
RYGB-6_mo
Pre-RYGB
NW
*
**
***

Supplementary Figure 2. Fecal microbial phylotypes that were enriched or depleted 12 months after RYGB surgery. *indicates statistical significance between pre-RYGB and RYGB-12_mo groups based on Wilcoxon signed-rank test and p values were corrected using Bonferroni method. *$p < 0.05$ and **$p < 0.01$.

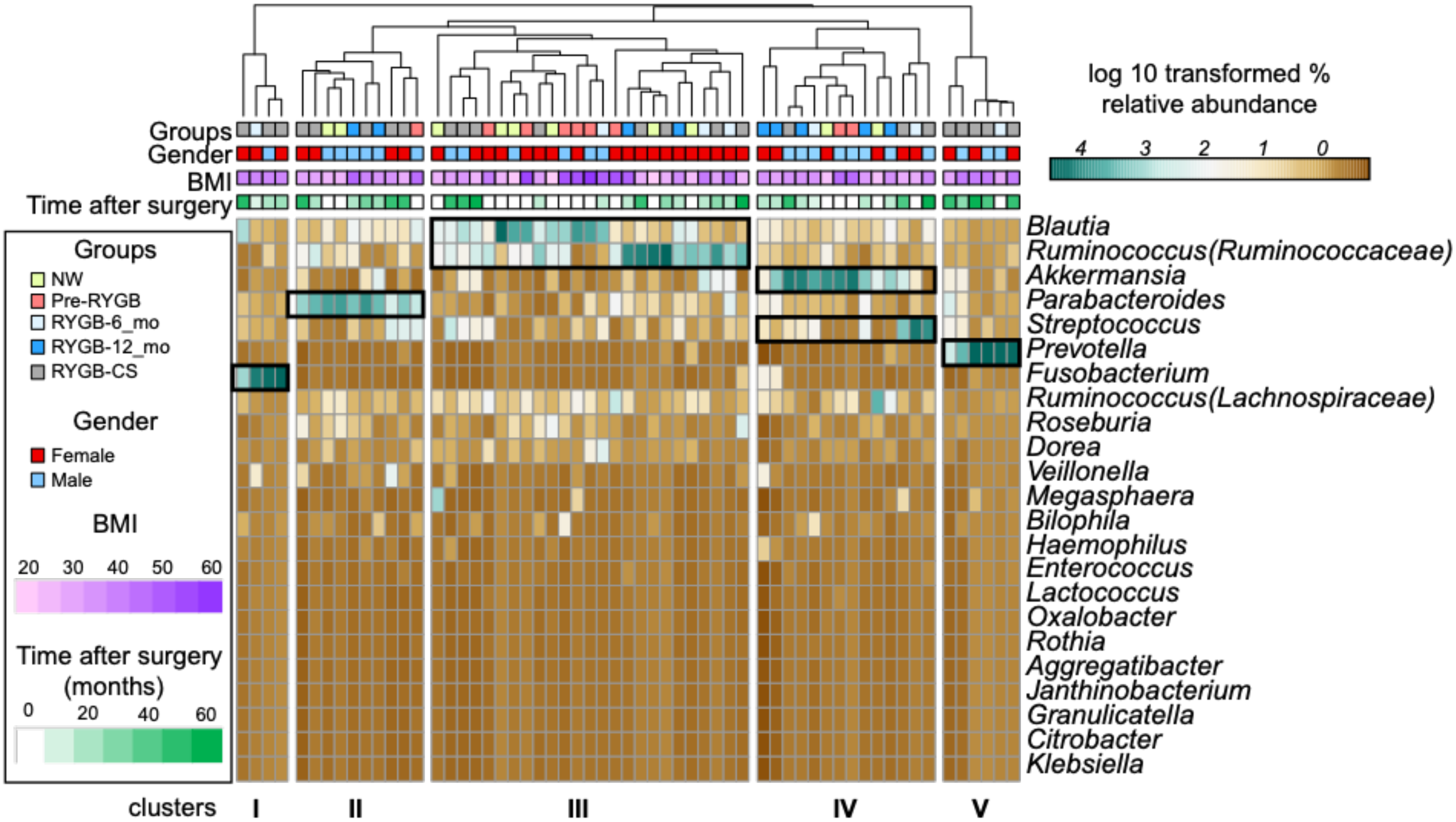

log 10 transformed % relative abundance
4
3
2
1
0
Groups
Gender
BMI
Time after surgery
Groups
NW
Pre-RYGB
RYGB-6_mo
RYGB-12_mo
RYGB-CS
Gender
Female
Male
BMI
20 30 40 50 60
Time after surgery (months)
0 20 40 60
Blautia
Ruminococcus(Ruminococcaceae)
Akkermansia
Parabacteroides
Streptococcus
Prevotella
Fusobacterium
Ruminococcus(Lachnospiraceae)
Roseburia
Dorea
Veillonella
Megasphaera
Bilophila
Haemophilus
Enterococcus
Lactococcus
Oxalobacter
Rothia
Aggregatibacter
Janthinobacterium
Granulicatella
Citrobacter
Klebsiella
clusters
I
II
III
IV
V

Supplementary Figure 3. Hierarchical cluster analysis of the fecal genus-level phylotypes based on Euclidean distances. The analysis was performed with ClustVis software. The clusters were driven by groups based on surgery (NW, Pre-RYGB, RYGB- 6 month, RYGB-12 month and RYGB-CS) more than gender, BMI or time after surgery. Samples formed 5 distinct clusters driven by *Fusobacterium, Prevotella*, *Ruminococcus*, *Parabacteroides*, *Blautia*, and *Akkermansia*. Three of the clusters were composed of only post-RYGB samples (RYGB-6_mo, RYGB_12-mo, and RYGB-CS), indicating the impact of the RYGB alone on the relative abundance of genus-level phylotypes.

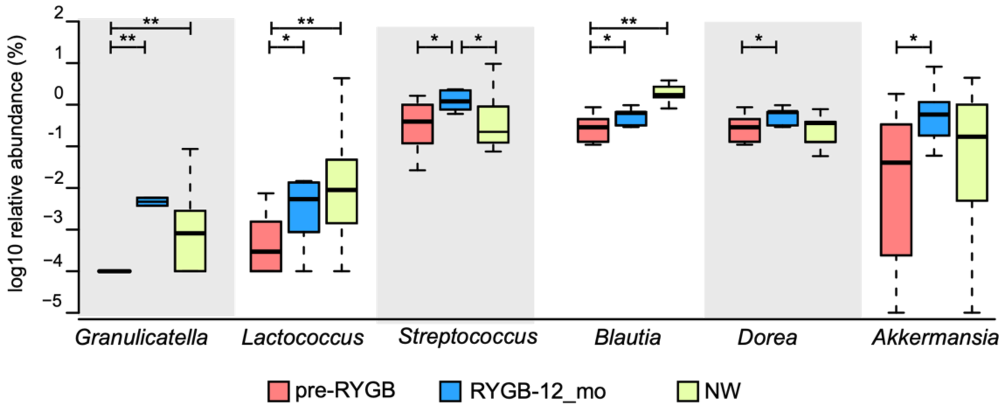

Supplementary Figure 4. Mucosal microbial phylotypes that were enriched or depleted 12 months after RYGB surgery. *indicates statistical significance between pre-RYGB and RYGB-12_mo groups based on Wilcoxon signed-rank test and p values were corrected using Bonferroni method. *$p < 0.05$ and **$p < 0.01$.

	HDCA	THDCA	GCDCA	UDCA	GUDCA	GDCA	TDCA	LCA	GLCA	TLCA	CA	TCA	GCA	TCDCA	HCA
Acetobacterium	0,3	0,1	0,5	0,5	0,3	0,7	0,5	0,3	0,0	0,3	0,2	0,1	0,5	0,2	-0,1
Achromobacter	-0,1	-0,4	0,2	-0,3	-0,3	0,6	0,0	0,0	-0,1	0,0	0,3	-0,3	0,4	-0,3	0,1
Acidovorax	0,0	-0,6	-0,4	-0,7	-0,6	0,0	-0,3	-0,1	0,1	-0,1	-0,1	-0,5	-0,2	-0,6	0,5
Agromyces	-0,1	-0,5	-0,2	-0,7	-0,6	0,2	-0,2	0,0	0,1	-0,1	0,1	-0,4	0,0	-0,5	0,3
Alcanivorax	-0,2	-0,6	-0,1	-0,6	-0,5	0,2	-0,3	-0,1	-0,1	-0,2	0,1	-0,4	0,1	-0,5	0,3
Anaerolinea	-0,1	-0,7	-0,2	-0,5	-0,6	0,3	0,0	0,0	-0,2	-0,1	0,0	-0,5	0,0	-0,5	0,4
Anaeromusa	0,3	0,3	0,6	0,4	0,5	0,7	0,5	0,1	0,1	0,4	0,3	0,3	0,6	0,5	-0,2
Bilophila	-0,1	-0,3	-0,5	0,0	-0,3	-0,4	-0,5	0,2	-0,1	0,1	-0,4	-0,4	-0,7	-0,3	0,0
Citrobacter	-0,1	0,0	0,4	0,1	0,2	0,6	0,1	-0,2	-0,3	-0,1	0,2	-0,1	0,4	0,1	0,1
Clostridium	0,5	0,1	0,5	0,0	0,0	0,8	0,7	0,1	0,3	0,6	0,4	0,3	0,5	0,3	0,1
Corynebacterium	0,2	0,1	0,3	0,3	0,1	0,6	0,4	0,6	0,4	0,5	0,2	0,0	0,2	0,1	-0,4
Desulfomicrobium	0,1	-0,6	-0,1	-0,6	-0,6	0,3	0,1	-0,2	0,0	0,0	0,2	-0,2	0,1	-0,3	0,5
Desulfovibrio	0,3	0,0	0,3	0,1	-0,2	0,5	0,6	0,5	0,5	0,5	0,2	0,1	0,1	0,1	-0,2
Lactobacillus	0,3	-0,1	0,2	-0,2	0,1	0,7	0,5	-0,1	0,2	0,4	0,1	0,1	0,4	0,1	0,0
Methanobacterium	0,3	-0,1	0,5	0,0	0,1	0,8	0,5	0,0	0,1	0,4	0,4	0,2	0,6	0,2	0,2
Methanocorpusculum	0,4	0,0	0,5	0,1	0,1	0,8	0,5	0,3	0,4	0,5	0,5	0,2	0,5	0,3	-0,1
Methylobacterium	-0,1	-0,6	-0,2	-0,3	-0,6	0,1	-0,2	0,2	0,1	-0,1	0,2	-0,5	-0,2	-0,4	0,1
Oxalobacter	0,0	-0,7	-0,5	-0,5	-0,7	-0,1	-0,3	0,0	-0,2	-0,2	-0,4	-0,6	-0,4	-0,7	0,5
Parvibaculum	-0,1	-0,5	-0,2	-0,7	-0,6	0,2	-0,2	0,0	0,1	-0,1	0,1	-0,4	0,0	-0,5	0,3
Sphingobium	-0,2	-0,6	-0,6	-0,8	-0,6	-0,1	-0,4	-0,1	0,1	-0,2	-0,3	-0,6	-0,4	-0,7	0,4
Sphingomonas	-0,2	-0,7	-0,2	-0,6	-0,6	0,3	-0,3	0,2	-0,1	-0,1	0,0	-0,6	-0,1	-0,6	0,0
Sutterella	-0,3	0,4	0,1	0,6	0,5	-0,4	-0,2	0,2	0,0	-0,3	0,1	0,2	0,0	0,2	-0,3
Treponema	0,3	0,0	0,5	-0,1	0,0	0,7	0,4	0,1	0,0	0,4	0,4	0,1	0,5	0,2	0,1
Turicibacter	0,3	0,2	0,4	0,3	0,3	0,5	0,4	0,1	0,0	0,3	0,0	0,1	0,4	0,2	0,2
Veillonella	-0,2	0,5	0,1	0,3	0,6	-0,4	0,0	-0,5	-0,3	-0,2	-0,2	0,4	0,2	0,3	-0,1

Supplementary Figure 5. Spearman's rank correlation coefficients based on concentration of bile acids and relative abundance of genus-level phylotypes from the mucosal samples. The framed boxes represent statistically significant associations. GUDCA, UDCA, and GDCA were the bile acids that correlated with the greatest number of phylotypes.

Supplementary Tables

Table S1. GC-MS analysis of fecal metabolites in NW, pre-RYGB, RYGB-6_mo, and RYGB-12_mo subject groups. Log2 transformed median concentrations and median absolute deviation values are reported.

	Pre-RYGB	RYGB - 6_mo	RYGB-12_mo	RYGB-CS	NW	Pre-RYGB vs RYGB-6_mo	Pre-RYGB vs RYGB-12_mo
	Median ± MAD					Wilcoxon signed-rank test p value	
**l-leucine**	3.7±0.7	1.6±0.2	1.5±0.2	2.8±1.0	3.7±0.5	0.000	0.000
**l-methionine**	0.4±0.8	-1.5±0.4	-1.6±0.5	-0.2±1.0	0.5±0.2	0.000	0.000
**l-threonine**	-1.1±0.4	-2.2±0.2	-2.3±0.1	-1.3±0.7	-1.2±0.5	0.001	0.000
**l-valine**	1.7±0.6	0.4±0.3	0.6±0.3	1.3±0.8	1.9±0.4	0.001	0.001
**l-alanine**	3.8±0.4	3.3±0.2	3.1±0.1	3.7±0.5	4.1±0.3	0.014	0.002
**l-isoleucine**	3.2±1.1	-0.3±1.5	-2.3±1.2	-1.2±3.2	-0.1±3.8	0.014	0.005

**dehydroalanine**	-2.4±0.3	-2.2±0.3	-2.0±0.3	-2.3±0.3	-2.3±0.3	0.079	0.002
**uracil**	2.1±0.5	1.5±0.3	1.2±0.3	2.0±0.6	2.5±0.4	0.120	0.003
**heptadecanoic acid**	2.8±0.6	4.0±0.5	4.6±0.9	4.0±0.6	3.3±0.3	0.002	0.000
**octadecenoic acid**	6.0±0.5	7.3±0.5	7.8±0.4	7.2±0.5	7.2±0.5	0.018	0.000
**oleanitrile**	0.6±0.3	1.4±0.5	1.5±0.4	0.9±0.4	0.8±0.3	0.079	0.000
**palmitic acid**	7.3±0.4	8.3±0.2	8.6±0.3	8.1±0.4	7.7±0.3	0.000	0.000
**stearic acid**	7.0±0.5	7.9±0.5	8.1±0.5	7.9±0.3	7.7±0.6	0.005	0.000
**pentadecanoic acid**	0.6±0.7	1.9±0.4	2.3±0.2	1.8±0.4	1.9±0.9	0.007	0.009
**arachidic acid**	2.6±0.6	4.5±0.8	4.8±0.6	4.2±0.7	3.4±0.4	0.000	0.002
**linoelaidic acid**	2.3±1.1	1.1±0.7	1.5±0.7	1.6±1.2	2.9±0.6	0.003	0.002
**heneicosanoic acid**	-0.9±0.4	0.5±0.7	0.3±0.4	-0.1±0.6	-0.4±0.2	0.002	0.003
**dodecanoic acid**	0.9±0.7	2.7±0.8	3.5±1.8	2.9±1.3	2.9±1.8	0.005	0.002
**oleamide**	2.2±0.4	2.9±0.4	3.0±0.4	3.310.6	3.2±0.4	0.024	0.003
**l-(+) lactic acid**	0.4±0.3	1.5±0.4	1.2±0.5	0.8±0.5	0.7±0.6	0.008	0.035

**pectin**	1.7±0.8	-0.7±0.7	-1.5±0.9	0.3±0.6	1.4±0.5	0.125	0.009
**d-xylose**	3.4±0.9	1.1±0.6	-0.8±1.4	2.2±0.9	3.0±0.5	0.290	0.056
**d-ribose**	4.1±0.3	3.1±0.5	2.1±1.0	3.9±0.6	4.2±1.0	0.052	0.003
**d-fructose**	2.1±0.7	0.1±0.5	-0.2±0.7	1.5±0.5	2.4±0.5	0.018	0.001
**d-glucose**	4.0±0.8	1.8±0.8	1.9±0.5	3.2±1.2	3.6±1.3	0.053	0.003

Table S2. Primary and secondary bile acids measured in the fecal matter from the participants (NW, RYGB-CS, RYGB-12_mo, and pre-RYGB groups). P stands for primary bile acids, and S stands for secondary bile acids. The concentrations are log2 transformed, median values along with median absolute deviation (MAD) values were reported.

**Bile acid type**	**Acronym**	**Type**	**NW**	**RYGB-CS**	**RYGB -12_mo**	**Pre-RYGB**	Pre-RYGB vs RYGB-6_mo	Pre-RYGB vs RYGB-12_mo
			Median ± MAD				Wilcoxon signed-rank test p value	
**Cholic acid**	CA	P	1.8±1.1	1.2±1.7	1.2±0.9	2.9±1.3	0.545	0.013
**Gylcochenodeoxy-cholic acid**	GCDCA	P	0.6±0.8	0.6±1.0	0.0±1.0	2.3±1.7	0.545	0.002
**Taurocholic acid**	TCA	P	-0.1±0.0	-1.5±1.3	-1.2±0.6	-0.1±0.3	0.570	0.054
**Glycocholic acid**	GCA	P	2.0±0.9	1.4±1.3	0.8±1.1	2.9±1.6	0.278	0.015
**Taurochenodeoxy-cholic acid**	TCDCA	P	-0.5±0.9	-1.9±1.2	-2.1±0.8	0.0±1.6	0.558	0.027

**Taurohydroxycholic acid**	THDCA	S	-5.8±1.7	-5.6±1.7	-5.3±2.1	-3.6±1.6	1	0.020
**Glycocholic acid**	GDCA	S	0.6±0.6	0.5±0.8	0.0±0.7	2.3±1.3	0.813	0.164
**Taurodeoxycholic acid**	TDCA	S	-0.9±1.4	-2.9±0.9	-2.6±0.5	0.2±08	0.737	0.008
**Lithocholic acid**	LCA	S	-0.5±1.2	-1.9±0.9	0.1±0.5	-1.6±0.4	0.725	0.027
**Glycolithocholic acid**	GLCA	S	-1.9±0.6	-1.2±0.9	-1.9±0.7	-0.5±0.3	0.558	0.005
**Taurolithocholic acid**	TLCA	S	-0.8±0.7	-1.1±1.0	-1.3±0.7	-0.1±0.8	0.292	0.110
**Hyocholic acid**	HCA	S	-1.0±0.9	-1.9±1.0	-1.8±0.7	-1.6±0.7	0.643	0.821
**Ursodeoxycholic acid**	UDCA	S	-1.1±1.1	-0.3±1.3	-0.8±0.8	0.9±1.4	0.485	0.472
**Glycoursodeoxycholic acid**	GUDCA	S	-3.9±1.1	-3.7±1.7	-4.1±2.3	-1.6±1.5	1	0.194
**Tauroursodeoxycholic acid**	TUDCA	S	-7.8±1.5	-7.3±2.4	-6.7±1.6	-6.5±2.8	0.318	0.751

Supplementary Methods

DNA extraction, 16S rRNA gene sequencing and analysis

We extracted microbial DNA from fecal and biopsy samples to represent fecal and mucosal microbiota. Samples were randomized before extraction. We used MOBIO PowerSoil DNA extraction kit (MOBIO Laboratories, Carlsbald, CA, USA) and followed the manufacturer's instructions. We prepared sequencing libraries using the protocols from Earth Microbiome project using V4 forward (GTGCCAGCMGCCGCGGTAA) and reverse (GGACTACHVGGGTWTCTAAT) primers with Illumina Miseq Instrument.[11] The sequences were deposited in the Sequence Read Archive (SRA) database (BioSample IDs = SAMN08684029-SAMN08684111). PANDAseq [2] paired reads were analyzed using QIIME 1.9 suite.[3] The analysis was followed by the following modification according to Kang *et al.*: clusters were formed at 99% sequence similarity in addition to 97% sequence similarity[4], and results presented were based on 99% sequence similarity. Sequencing errors were reduced by removing chimeric sequences and OTUs with low sequence abundance; OTUs that contain less than 0.005% of the total number of sequences were omitted from the analysis as previously recommended.[5] We calculated alpha and beta diversity metrics of Phylogenetic Diversity Whole Tree[6], and Unifrac[7] to better understand microbial community structures. Gene abundances for bile acid biosynthesis were predicted with Phylogenetic Investigation of Communities by Reconstruction of Unobserved Species (PICRUSt) software.[8] The details of the bioinformatic data analysis can be found in a previous publication.[9]

^{1}H-NMR analysis of water-soluble fecal metabolites

For each fecal specimen, approximately one gram of wet weight (precise weight was recorded and used for calculations) was diluted with 20 mL of milliQ water (18 ohms). This step was performed in triplicate. The sample was vortexed at the highest speed for three minutes to achieve complete mixing. Then, the homogenates were centrifuged at 16,110 x g for 15 minutes and the supernatants were filtered through 0.2-um PVDF membranes (PALL Corporation). The fecal extracts were diluted with a 10% (v/v) spike of a National Institute of Standards and Technology calibrated reference solution (100% D2O, 5 mM 2,2-dimethyl-2-silapentane-5-sulfonate-d6 (DSS), and 0.1% sodium azide). The resulting mixture was loaded into 3-mm NMR tubes (Bruker Inc) and shipped to PNNL on cold-packs for NMR analysis. All NMR spectra were collected using a Varian Direct Drive 600 MHz NMR spectrometer equipped with a 5-mm triple-resonance salt-tolerant cold probe. The 1D ^{1}H-NMR spectra of all samples were processed, assigned, and analyzed by using Chenomx NMR Suite 8.1 with quantification of metabolites based on spectral intensities relative to the internal standard and as previously described.[9]

Liquid chromatography-mass spectrometry analysis of fecal bile acids

Fecal samples were lyophilized before extraction. 50 µL of internal standard mixture (1.0 µg/mL) was spiked into 5 mg of lyophilized stool samples and the samples were incubated with 1.8 mL of 0.1 M NaOH at 60°C on a thermomixer with shaking at 1200 rpm. Then, the whole samples were transferred to 15-mL centrifuge tubes containing 2.0 mL of ultrapure (Milli-Q) water. An additional 2.0 mL of water was used to rinse each tube and combine with the appropriate sample in the 15-mL tube.

Samples were then homogenized using an OMNI homogenizer, changing the tip between samples. Then samples were centrifuged at 13,600 x g for 20 minutes and the supernatant was filtered using a 25 mm Acrodisc GHP 45 µm syringe-filter. All samples were cleaned-up using a 60 mg Oasis HLB 3cc cartridge (Waters Corporation, Milford, MA), dried in vacuo, and stored at-70°C until analysis.

Bile acid standards were purchased from Steraloids Inc. (Newport, RI) and Sigma-Aldrich (St. Louis, MO). Optima grade methanol, acetonitrile and formic acid were purchased from Fisher Scientific (Hampton, NH). The internal standard (23-nor-5β-cholanic acid-3α, 12α-diol, IS) was purchased from Steraloids Inc. (Newport, RI). Purified, deionized water, >18 MΩ, (Nanopure Infinity ultrapure water system, Barnstead, Newton, WA) was used to make all HPLC and sample solvents. The sample preparation and bile acid quantification procedures were based on the method of Humbert et al, with modification.[10] Stock solutions of individual bile acids were made in methanol (1 mg/mL) and stored at -20˚C. Aliquots of each bile acid were then pooled together, dried, and serially diluted in nanopure water to make a calibration curve (1.5 ng/mL, 3.1 ng/mL, 6.3 ng/mL, 13 ng/mL, 25 ng/mL, 50 ng/mL, 100 ng/mL, 500 ng/mL, 1 µg/mL, 5 µg/mL, 10 µg/mL). Calibration curves were created by plotting the ratios of each bile acid peak area to the internal standard peak area as a function of the respective standard concentration.

A Waters Nano-Acquity UPLC system (Waters Corporation, Milford, MA) was configured for direct 5-µL injections of samples onto an in-house packed, fused silica column (360 µm o.d. x 150µm i.d. x 30 cm long; Polymicro Technologies Inc., Phoenix, AZ) containing HSS T3 reversed-phase media (1.8 µm; Waters Corporation) and at a

flow rate of 600 nL/min. Mobile phases consisted of (A) 0.1% formic acid in water and (B) 0.12 % formic acid and 5 mM ammonium acetate in methanol. The gradient profile was (min, %B): 0, 1; 5, 1; 10, 65; 59, 99; and 60, 1. Total run time, including column re-equilibration, was 75 min. MS analysis was performed using an Agilent model 6490 triple quadrupole mass spectrometer (Agilent Technologies, Santa Clara, CA) outfitted with an in-house nano-electrospray ionization interface. Electrospray emitters were constructed in-house using 150 μm o.d. x 20 μm i.d. chemically etched fused silica [11]. The hexabore ion transfer tube temperature and spray voltage were held at 200 ˚C and -4.0 kV, respectively. Data were acquired in negative-ion mode using selected reaction monitoring (SRM) for 75 minutes from sample injection using a dwell time of 200 μs, fragmentation of 380 volts, and collision energy of 10 volts.

Gas chromatography-mass spectrometry analysis of fecal metabolites

Metabolites were extracted from 10 mg of lyophilized stool samples using methanol with sonication. Extracted metabolites were completely dried *in vacuo* and chemically derivatized as reported previously.[12] Briefly, the extracted metabolites were derivatized by methoxyamination and trimethylsilyation (TMS), then the samples were analyzed by GC-MS. GC-MS raw data files were processed using the Metabolite Detector software, version 2.5 beta.[13] Briefly, Agilent D files were converted to netCDF format using Agilent Chemstation, followed by conversion to binary files using Metabolite Detector. Retention indices (RI) of detected metabolites were calculated based on the analysis of a FAMEs mixture, followed by their chromatographic alignment across all analyses after deconvolution. Metabolites were initially identified by matching experimental spectra to an augmented version of FiehnLib (Retention time Locked

(RTL)). Library, containing spectra and validated retention indices for over 850 metabolites), using Metabolite Detector, then unknown peaks were additionally matched with the NIST14 GC-MS library. All metabolite identifications were manually validated to reduce deconvolution errors during automated data-processing and to eliminate false identifications. All raw GC-MS data will be made available via the MetaboLights metabolomics data repository (http://www.ebi.ac.uk/metabolights/index).

Supplementary References

1. Gilbert, J. A., Jansson, J. K. & Knight, R. The Earth Microbiome project: Successes and aspirations. *BMC Biology* vol. 12 (2014).
2. Masella, A. P., Bartram, A. K., Truszkowski, J. M., Brown, D. G. & Neufeld, J. D. PANDAseq: Paired-end assembler for illumina sequences. *BMC Bioinformatics* **13**, (2012).
3. Caporaso, J. G. *et al.* QIIME allows analysis of high-throughput community sequencing data. *Nature Methods* vol. 7 335–336 (2010).
4. Kang, D.-W. *et al.* Gut microbial and short-chain fatty acid profiles in adults with chronic constipation before and after treatment with lubiprostone. *Anaerobe* **33**, 33–41 (2015).
5. Mcmurdie, P. J. *et al.* Advancing our understanding of the human microbiome using QIIME. *Methods Enzymol.* **531**, 371–444 (2015).
6. Faith, D. P. Conservation evaluation and phylogenetic diversity. *Biol. Conserv.* **61**, 1–10 (1992).
7. Lozupone, C., Hamady, M. & Knight, R. UniFrac - An online tool for comparing microbial community diversity in a phylogenetic context. *BMC Bioinformatics* **7**,

(2006).

8. Langille, M. G. I. *et al.* Predictive functional profiling of microbial communities using 16S rRNA marker gene sequences. *Nat. Biotechnol.* **31**, 814–821 (2013).

9. Ilhan, Z. E. *et al.* Distinctive microbiomes and metabolites linked with weight loss after gastric bypass, but not gastric banding. vol. 11 1–12 (2017).

10. Humbert, L. *et al.* Bile acid profiling in human biological samples: Comparison of extraction procedures and application to normal and cholestatic patients. *J. Chromatogr. B Anal. Technol. Biomed. Life Sci.* **899**, 135–145 (2012).

11. Kelly, R. T. *et al.* Chemically etched open tubular and monolithic emitters for nanoelectrospray ionization mass spectrometry. *Anal. Chem.* **78**, 7796–7801 (2006).

12. Snijders, A. M. *et al.* Influence of early life exposure, host genetics and diet on the mouse gut microbiome and metabolome. *Nat. Microbiol.* **2**, 16221 (2016).

13. Hiller, K. *et al.* Metabolite detector: Comprehensive analysis tool for targeted and nontargeted GC/MS based metabolome analysis. *Anal. Chem.* **81**, 3429–3439 (2009).
